# Supplementary material for: Comparative efficacy of terlipressin and norepinephrine for treatment of hepatorenal syndrome-acute kidney injury: A systematic review and meta-analysis
Source: PLoS One. 2024 Jan 29;19(1):e0296690. doi: 10.1371/journal.pone.0296690 (PMC10824429; doi:10.1371/journal.pone.0296690)
Supplement: S1 Table — (DOCX) [file pone.0296690.s003.docx]

**SUPPLEMENTAL INFORMATION**

**Supplementary Table S1. Search strategies and selection criteria for studies.**

| PubMed Search Results | | | | |
| --- | --- | --- | --- | --- |
| MeSH terms: “hepatorenal syndrome” OR “hepatorenal syndrome-acute kidney injury” AND “terlipressin” AND “norepinephrine” OR “noradrenaline”  (“hepatorenal syndrome”[MeSH Terms]) OR (“hepatorenal syndrome-acute kidney injury”[MeSH Terms]) AND (“terlipressin”[MeSH Terms]) AND (“norepinephrine”[MeSH Terms]) OR (“noradrenaline”[MeSH Terms])  Limits: 2007-2023, Humans, English | | | | |
| Article | Year | First author | Inclusion/exclusion | Reason for inclusion or exclusion |
| 1. Terlipressin versus other vasoactive drugs for hepatorenal syndrome | 2017 | Israelsen M | Excluded | Meta-analysis |
| 1. The comparative effectiveness of vasoactive treatments for hepatorenal syndrome: a systematic review and network meta-analysis | 2022 | Pitre T | Excluded | Meta-analysis |
| 1. Terlipressin versus noradrenaline in the treatment of hepatorenal syndrome: systematic review with meta-analysis and full economic evaluation | 2016 | Mattos AZ | Excluded | Meta-analysis |
| 1. Hepatorenal syndrome: current concepts related to diagnosis and management | 2016 | de Mattos AZ | Excluded | Not a randomized controlled trial (RCT) |
| 1. Hepatorenal syndrome: the clinical impact of vasoactive therapy | 2018 | Colle I | Excluded | Not an RCT |
| 1. Toward norepinephrine as a first-line treatment for all hospitalized patients with hepatorenal syndrome | 2021 | Paine CH | Excluded | Not an RCT |
| 1. Efficacy and safety of terlipressin in cirrhotic patients with variceal bleeding or hepatorenal syndrome | 2008 | Krag A | Excluded | Does not evaluate comparative efficacy of terlipressin or norepinephrine |
| 1. Noradrenaline or terlipressin for hepatorenal syndrome? | 2015 | Celis P | Excluded | Not an RCT |
| 1. Cost-effectiveness of using terlipressin to treat hepatorenal syndrome | 2022 | Ferreira LM | Excluded | Meta-analysis; focus on cost |
| 1. Recent advances in our understanding of hepatorenal syndrome | 2012 | Wong F | Excluded | Not an RCT |
| 1. Terlipressin versus norepinephrine in the treatment of hepatorenal syndrome: a systematic review and meta-analysis | 2014 | Nassar AP Jr | Excluded | Meta-analysis |
| 1. Noradrenaline vs terlipressin in the treatment of hepatorenal syndrome: a randomized study | 2012 | Singh V | Included | Compares efficacy of terlipressin and norepinephrine amongst patients with hepatorenal syndrome type 1 (HRS-1) with HRS reversal as a primary endpoint |
| 1. Emerging hepatic syndromes: pathophysiology, diagnosis, and treatment | 2016 | Bertino G | Excluded | Not an RCT |
| 1. Noradrenaline versus terlipressin in the management of type 1 hepatorenal syndrome: a randomized controlled study | 2018 | Saif RU | Included | Compares efficacy of terlipressin and norepinephrine amongst patients with HRS-1 with HRS reversal as a primary endpoint |
| 1. Noradrenaline is as effective as terlipressin in hepatorenal syndrome type 1: a prospective, randomized trial | 2016 | Goyal O | Included | Compares efficacy of terlipressin and norepinephrine amongst patients with HRS-1 with HRS reversal as a primary endpoint |
| 1. Comparative efficacy of pharmacological strategies for management of type 1 hepatorenal syndrome: a systematic review and network meta-analysis | 2017 | Facciorusso A | Excluded | Meta-analysis |
| 1. Comparative efficacy of vasoconstrictor therapies for type 1 hepatorenal syndrome: a network meta-analysis | 2017 | Zheng JN | Excluded | Meta-analysis |
| 1. Noradrenaline vs terlipressin in the treatment of type 2 hepatorenal syndrome: a randomized pilot study | 2013 | Ghosh S | Excluded | Does not include patients with HRS-1 |
| 1. Terlipressin versus noradrenaline for hepatorenal syndrome. Economic evaluation under the perspective of the Brazilian public health system | 2016 | Mattos AZ | Excluded | Does not evaluate HRS reversal as an endpoint |
| 1. Limited progress in hepatorenal syndrome (HRS) reversal and survival 2002-2018: a systematic review and meta-analysis | 2020 | Thomson MJ | Excluded | Meta-analysis |
| 1. Prevalence and short-term outcome of hepatorenal syndrome: a 9-year experience in a high-complexity hospital in Colombia | 2020 | Rey R M | Excluded | Retrospective study; not an RCT |
| 1. The efficacy and safety of terlipressin and albumin in patients with type 1 hepatorenal syndrome: a multicenter, open-label, explorative study | 2012 | Narahara Y | Excluded | Does not evaluate comparative efficacy of terlipressin and norepinephrine |
| 1. New insights into the management of hepatorenal syndrome | 2011 | Hasper D | Excluded | Not an RCT |
| 1. Noradrenalin vs terlipressin in patients with hepatorenal syndrome: a prospective, randomized, unblinded, pilot study | 2007 | Alessandria C | Included | Compares efficacy of terlipressin and norepinephrine amongst patients with HRS-1 with HRS reversal as a primary endpoint |
| 1. An open-label, pilot, randomized controlled trial of noradrenaline versus terlipressin in the treatment of type 1 hepatorenal syndrome and predictors of response | 2008 | Sharma P | Included | Compares efficacy of terlipressin and norepinephrine amongst patients with HRS-1 with HRS reversal as a primary endpoint |
| 1. Noradrenaline for hepatorenal syndrome in patients with acute-on-chronic liver failure: hope remains! | 2018 | Satsangi S | Excluded | Not an RCT |
| 1. Noradrenaline as an alternative medical treatment to terlipressin in the management of hepatorenal syndrome type 1 | 2018 | Sendra C | Excluded | Not an RCT |
| 1. Pharmacological treatment of hepatorenal syndrome: a note of optimism | 2007 | Salerno F | Excluded | Not an RCT |
| 1. Meta-analysis of terlipressin in treatment of hepatorenal syndrome: an update | 2009 | Zhang ZF | Excluded | Meta-analysis |

| Cochrane Library Results | | | | |
| --- | --- | --- | --- | --- |
| Keyword: Terlipressin  Title abstract keyword: “norepinephrine” OR “noradrenaline” AND “hepatorenal syndrome” OR “hepatorenal syndrome-acute kidney injury”  Limits: 2007-2022 | | | | |
| Title | Year | Author or trial identifier | Inclusion/exclusion | Reason for exclusion or inclusion |
| 1. Type 2 hepatorenal syndrome | 2012 | NCT01637454 | Excluded | Does not include patients with  HRS-1 |
| 1. Terlipressin infusion alone vs terlipressin with noradrenaline infusion in the treatment of hepatorenal syndrome type 1 | 2019 | NCT03822091 | Excluded | Does not evaluate comparative efficacy of terlipressin vs norepinephrine in patients with HRS |
| 1. Noradrenaline vs terlipressin in the treatment of hepatorenal syndrome: a randomized study | 2012 | Singh V | Included | Compares efficacy of terlipressin and norepinephrine amongst patients with HRS-1 with HRS reversal as a primary endpoint |
| 1. Noradrenaline versus terlipressin in the management of type 1 hepatorenal syndrome: a randomized controlled study | 2018 | Saif RU | Included | Compares efficacy of terlipressin and norepinephrine amongst patients with HRS-1 with HRS reversal as a primary endpoint |
| 1. To compare the response rate of noradrenaline vs terlipressin in hepatorenal syndrome in patients with acute-on-chronic liver failure | 2015 | NCT02573727 | Included | This is the registration of the Arora trial included in the analysis |
| 1. Noradrenaline vs terlipressin in the treatment of type 2 hepatorenal syndrome: a randomized pilot study | 2013 | Ghosh S | Excluded | Only includes patients with  HRS type 2 |
| 1. Meta-analysis of terlipressin in treatment of hepatorenal syndrome: an update | 2009 | Zhang ZF | Excluded | Meta-analysis |
| 1. Noradrenaline is as effective as terlipressin in hepatorenal syndrome type 1: a prospective, randomized trial | 2016 | Goyal O | Included | Compares efficacy of terlipressin and norepinephrine amongst patients with HRS-1 with HRS reversal as a primary endpoint |
| 1. Noradrenalin vs terlipressin in patients with hepatorenal syndrome: a prospective, randomized, unblinded, pilot study | 2007 | Alessandria C | Included | Compares efficacy of terlipressin and norepinephrine amongst patients with HRS-1 with HRS reversal as a primary endpoint |
| 1. An open-label, pilot, randomized controlled trial of noradrenaline versus terlipressin in the treatment of type 1 hepatorenal syndrome and predictors of response | 2008 | Sharma P | Included | Compares efficacy of terlipressin and norepinephrine amongst patients with HRS-1 with HRS reversal as a primary endpoint |
| 1. Terlipressin is superior to noradrenaline in the management of acute kidney injury in acute-on-chronic liver failure | 2020 | Arora V | Included | Compares efficacy of terlipressin and norepinephrine amongst patients with HRS-1 with HRS reversal as a primary endpoint |
| 1. A study of OCE-205 in participants with cirrhosis with ascites who developed hepatorenal syndrome-acute kidney injury | 2022 | NCT05309200 | Excluded | Does not evaluate comparative efficacy of terlipressin and norepinephrine |
| 1. Norepinephrine is more effective than midodrine/octreotide in patients with hepatorenal syndrome-acute kidney injury: a randomized controlled trial | 2021 | El El-Desoki M | Excluded | Does not evaluate comparative efficacy of terlipressin and norepinephrine |
| 1. Noradrenalin versus midodrine-octreotide in hepatorenal syndrome | 2011 | IRCT201107217085N1 | Excluded | Does not evaluate comparative efficacy of terlipressin and norepinephrine |
| 1. Noradrenaline versus terlipressin in the treatment of hepatorenal syndrome | 2010 | Singh V | Excluded | Abstract of the Singh paper |
| 1. Noradrenaline versus terlipressin in the treatment of hepatorenal syndrome | 2011 | Singh V | Excluded | Abstract of the Singh paper |
| 1. Respiratory events with terlipressin and albumin in hepatorenal syndrome: a review and clinical guidance | 2022 | Allegretti AS | Excluded | Not an RCT |
| 1. Noradrenaline versus terlipressin in the treatment of hepatorenal syndrome: a randomized study | 2022 | Koneti A | Excluded | Patients received albumin and midodrine along with terlipressin and norepinephrine |
| 1. Combination of terlipressin and noradrenaline in non-responders of terlipressin infusion in hepatorenal syndrome | 2019 | Jayachandran A | Excluded | Evaluates patients who did not respond to terlipressin |
| 1. Noradrenalin versus the combination of midodrine and octreotide in patients with hepatorenal syndrome: randomized clinical trial | 2012 | Tavakkoli H | Excluded | Does not evaluate comparative efficacy of terlipressin and norepinephrine |
| 1. Terlipressin versus noradrenalin in hepatorenal syndrome: a prospective, randomized, unblinded study | 2008 | Goyal O | Excluded | Most likely an abstract of the Goyal study |
| 1. Noradrenaline versus glypressin for prevention of hypotension after deflation of tourniquet in knee arthroplasty | 2023 | NCT05774067 | Excluded | Does not evaluate comparative efficacy of and norepinephrine amongst patients with HRS-1 |
| 1. Optimal timing of renal replacement therapy initiation in acute kidney injury: the elephant felt by the blindmen? | 2017 | Shiao CC | Excluded | Does not evaluate comparative efficacy of terlipressin and norepinephrine |
| 1. Noradrenaline is equally effective as terlipressin in reversal of type 1 hepatorenal syndrome: a randomized prospective study | 2013 | Indrabi RA | Included | Compares efficacy of terlipressin and norepinephrine amongst patients with HRS-1 with HRS reversal as a primary endpoint |
| 1. Comparison of noradrenaline with the combination of octreotide and midodrine in the treatment of hepatorenal syndrome type 1 | 2023 | IRCT2023022805768N1 | Excluded | Does not evaluate comparative efficacy of and norepinephrine |
| 1. Clinical trial comparing noradrenaline (NA) plus placebo versus noradrenaline plus terlipressin (TP) in septic shock | 2022 | NCT05207280 | Excluded | Does not include patients with HRS |
| 1. A comparative assessment of norepinephrine and terlipressin in the management of hepatorenal syndrome | 2022 | Jha UC | Excluded | Does not evaluate HRS reversal as an endpoint |
| 1. Extracorporeal liver support therapy with Prometheus in patients with hepatorenal syndrome | 2010 | DRKS00000441 | Excluded | Does not evaluate comparative efficacy of terlipressin and norepinephrine |
| 1. Continuous terlipressin infusion in septic shock | 2012 | NCT01697410 | Excluded | Does not include patients with HRS |
| 1. Evaluation of early association of terlipressin and norepinephrine during septic shock; the TerliNor study | 2017 | NCT03336814 | Excluded | Does not include patients with HRS |
| 1. The effect of terlipressin on intestinal function in septic shock patients | 2014 | NCT02306239 | Excluded | Does not include patients with HRS |
| 1. Terlipressin alone versus the standard therapy with catecholamines for hepatic patients with septic shock – prospective single-center randomized controlled study | 2018 | NCT03608514 | Excluded | Does not include patients with HRS |
| 1. Continuous terlipressin infusion in septic shock | 2012 | NCT01697410 | Excluded | Does not include patients with HRS |
| 1. Microcirculation recruitment using albumin 20% and terlipressin in septic patients | 2021 | NCT05080543 | Excluded | Does not include patients with HRS |
| 1. Terlipressin is superior to noradrenaline in management of acute kidney injury in acute-on-chronic liver failure | 2017 | Arora V | Excluded | Abstract of the Arora study |
| 1. Terlipressin is superior to noradrenaline in the management of acute kidney injury (AKI) in patients with ACLF | 2017 | Arora V | Excluded | Abstract of the Arora study |
| 1. To compare the efficacy of noradrenaline and terlipressin vs step-up terlipressin therapy in hepatorenal syndrome | 2012 | NCT01649037 | Excluded | Evaluates norepinephrine in combination with terlipressin |
| 1. Hypertonic saline and terlipressin for sepsis-associated hypotension | 2011 | NCT01271114 | Excluded | Does not include patients with HRS |
| 1. Terlipressin for refractory septic shock | 2020 | NCT04339868 | Excluded | Does not include patients with HRS |
| 1. Continuous infusion of terlipressin in septic shock | 2007 | NCT00481572 | Excluded | Does not include patients with HRS |
| 1. Efficacy and safety of monotherapy with noradrenaline and terlipressin in patients of cirrhosis with septic shock admitted to intensive care unit | 2013 | NCT01836224 | Excluded | Does not include patients with HRS |
| 1. Albumin administration in the prevention of hepatorenal syndrome and death in patients with cirrhosis, bacterial infections other than spontaneous bacterial peritonitis, and high risk of hospital mortality | 2014 | EUCTR2013-002416-27-AT | Excluded | Does not evaluate comparative efficacy of terlipressin and norepinephrine in patients with HRS |
| 1. A prospective, open-label, randomized noninferiority trial to compare the efficacy and safety of monotherapy with noradrenaline and terlipressin in patients of cirrhosis with septic shock admitted to the intensive care unit (NCT01836224) | 2014 | Choudhury AK | Excluded | Does not include patients with HRS |
| 1. Addition of terlipressin to norepinephrine in septic shock and effect of renal perfusion: a pilot study | 2022 | Wang J | Excluded | Does not include patients with HRS |
| 1. Terlipressin in septic shock in cirrhosis | 2008 | NCT00628160 | Excluded | Does not include patients with HRS |
| 1. To assess the efficacy of early introduction of a combination of low dose vasopressin analogue in addition to noradrenaline as a vasopressor in patients of cirrhosis with septic shock | 2015 | NCT02468063 | Excluded | Does not include patients with HRS |
| 1. Vasopressors to treat refractory septic shock | 2020 | Meresse Z | Excluded | Does not include patients with HRS |
| 1. Terlipressin versus norepinephrine as infusion in patients with septic shock: a multicentre, randomised, double-blinded trial | 2018 | Liu ZM | Excluded | Does not include patients with HRS |
| 1. Comparison of effect of norepinephrine and terlipressin on patients with ARDS combined with septic shock: a prospective, single-blind, randomized controlled trial | 2017 | Chen Z | Excluded | Does not include patients with HRS |
| 1. Terlipressin in septic shock: effects on microcirculation | 2009 | NCT00995839 | Excluded | Does not include patients with HRS |
| 1. Terlipressin versus norepinephrine to prevent milrinone-induced systemic vascular hypotension in cardiac surgery patient with pulmonary hypertension | 2019 | Abdelazziz MM | Excluded | Does not include patients with HRS |
| 1. Effects of terlipressin on patients with sepsis via improving tissue blood flow | 2016 | Xiao X | Excluded | Does not include patients with HRS |
| 1. Effects of vasopressinergic receptor agonists on sublingual microcirculation in norepinephrine-dependent septic shock | 2011 | Morelli A | Excluded | Does not include patients with HRS |
| 1. Comparison of effect of norepinephrine and terlipressin on patients with ARDS combined with septic shock: a prospective, single-blind, randomized controlled trial | 2017 | Zhi C | Excluded | Does not include patients with HRS |
| 1. Continuous terlipressin versus vasopressin infusion in septic shock (TERLIVAP): a randomized, controlled pilot study | 2009 | Morelli A | Excluded | Does not include patients with HRS |
| 1. Terlipressin in the treatment of late phase catecholamine-resistant septic shock | 2012 | Svoboda P | Excluded | Does not include patients with HRS |
| 1. A randomized trial comparing terlipressin and noradrenaline in patients with cirrhosis and septic shock | 2017 | Choudhury A | Excluded | Does not include patients with HRS |
| 1. Decision support for intraoperative low blood pressure | 2016 | NCT02726620 | Excluded | Does not evaluate comparative efficacy of terlipressin and norepinephrine in patients with HRS |
| 1. RaGuS trial by postoperative patients | 2020 | NCT04440085 | Excluded | Does not evaluate comparative efficacy of terlipressin and norepinephrine in patients with HRS |
| 1. Study to evaluate if the drug vasopressin protects the kidneys for patients undergoing liver transplant | 2009 | NCT00886262 | Excluded | Does not evaluate comparative efficacy of terlipressin and norepinephrine in patients with HRS |
| 1. Methylene blue versus vasopressin analog for refractory septic shock in the preterm neonate: a randomized controlled trial | 2022 | Ismail R | Excluded | Does not evaluate comparative efficacy of terlipressin and norepinephrine in patients with HRS |
| 1. Effects of vasopressinergic V1 receptor agonists on sublingual microcirculatory blood flow in patients with catecholamine-dependent septic shock | 2011 | Morelli A | Excluded | Does not include patients with HRS |
| 1. Effects of short-term simultaneous infusion of dobutamine and terlipressin in patients with septic shock: the DOBUPRESS study | 2008 | Morelli A | Excluded | Does not include patients with HRS |
| 1. Efficacy and safety of terlipressin in cirrhotic patients with variceal bleeding or hepatorenal syndrome | 2008 | Krag A | Excluded | Does not evaluate comparative efficacy of terlipressin and norepinephrine |
| 1. Hypertonic saline improves antidiuretic hormone levels in the presence of a terlipressin drip without apparent clinical benefits | 2015 | Pascual-Ramirez J | Excluded | Does not evaluate comparative efficacy of terlipressin and norepinephrine in patients with HRS |
| 1. Impact of terlipressin infusion during and after live donor liver transplantation on incidence of acute kidney injury and neutrophil gelatinase-associated lipocalin serum levels: a randomized controlled trial | 2017 | Kandil MA | Excluded | Does not evaluate comparative efficacy of terlipressin and norepinephrine in patients with HRS |
| 1. Terlipressin improves renal function in patients with cirrhosis and ascites without hepatorenal syndrome | 2007 | Krag A | Excluded | Does not include patients with HRS |
| 1. The INFECIR-2 albumin prevention study | 2014 | NCT02034279 | Excluded | Does not evaluate comparative efficacy of terlipressin and norepinephrine in patients with HRS |
| 1. A study of the heart and kidney function during terlipressin and dobutamine treatment in patients with chronic liver disease (cirrhosis) and fluid retention (ascites) | 2014 | EUCTR2012-002275-33-DK | Excluded | Does not evaluate comparative efficacy of terlipressin and norepinephrine in patients with HRS |
| 1. Liver-HERO: hepatorenal syndrome-acute kidney injury (HRS-AKI) treatment with transjugular intrahepatic portosystemic shunt in patients with cirrhosis a randomized controlled trial | 2023 | Ripoll C | Excluded | Does not evaluate comparative efficacy of terlipressin and norepinephrine in patients with HRS |

| ResearchGate Search Results | | | | |
| --- | --- | --- | --- | --- |
| Keywords: “hepatorenal syndrome” OR “hepatorenal syndrome-acute kidney injury” AND “terlipressin” AND “norepinephrine” OR “noradrenaline” | | | | |
| Article | Year | First author | Inclusion/exclusion | Reason for exclusion or inclusion |
| 1. Vasoactive drugs and acute kidney injury | 2008 | Bellomo R | Excluded | Does not evaluate comparative efficacy of terlipressin and norepinephrine |
| 1. Hepatorenal syndrome with acute kidney injury: diagnosis and medical management | 2023 | Erstad BL | Excluded | Not an RCT |
| 1. European internal medicine professionals hepatorenal syndrome: a mini review | 2023 | Bulur A | Excluded | Not an RCT |
| 1. Modified treatment of hepatorenal syndrome type I depending on the stage of acute kidney injury | 2021 | Slyvka NO | Excluded | Does not evaluate comparative efficacy of terlipressin and norepinephrine |
| 1. Comparative efficacy of terlipressin and norepinephrine for treatment of hepatorenal syndrome-acute kidney injury (HRS-AKI): a systematic review and meta-analysis | 2022 | Olson JC | Excluded | Preprint not published |
| 1. Symptomatic hyponatremia in a patient with hepatorenal syndrome treated with terlipressin | 2022 | Ahmed R | Excluded | Does not evaluate comparative efficacy of terlipressin and norepinephrine |
| 1. Cost-effectiveness of using terlipressin to treat hepatorenal syndrome | 2022 | Ferreira LM | Excluded | Meta-analysis; focus on cost |
| 1. Terlipressin for hepatorenal syndrome | 2012 | Krag A | Excluded | Only evaluates terlipressin for HRS patients |
| 1. Noradrenaline vs terlipressin for hepatorenal syndrome (no to hepatorenal syndrome): a meta-analysis | 2015 | Pedracio FHLP | Excluded | Meta-analysis |
| 1. Terlipressin versus other vasoactive drugs for hepatorenal syndrome | 2017 | Israelsen M | Excluded | Meta-analysis |
| 1. Terlipressin in the treatment of hepatorenal syndrome: a systematic review and meta-analysis | 2018 | Wang H | Excluded | Meta-analysis |
| 1. Real‐world treatment patterns and outcomes using terlipressin in 203 patients with hepatorenal syndrome | 2020 | Khurram J | Excluded | Does not evaluate comparative efficacy of terlipressin and norepinephrine |
| 1. Hepatorenal syndrome | 2019 | Francoz C | Excluded | Not an RCT |
| 1. Norepinephrine is more effective than midodrine/octreotide in patients with hepatorenal syndrome-acute kidney injury: a randomized controlled trial | 2021 | El-Desoki M | Excluded | Does not evaluate comparative efficacy of terlipressin and norepinephrine |
| 1. An integrated review of hepatorenal syndrome | 2021 | Ojeda-Yuren AS | Excluded | Does not evaluate comparative efficacy of terlipressin and norepinephrine |
| 1. Management of hepatorenal syndrome in liver cirrhosis: a recent update | 2022 | Bera C | Excluded | Not an RCT |
| 1. Combination of terlipressin and noradrenaline versus terlipressin in hepatorenal syndrome with early non-response to terlipressin infusion: a randomized trial | 2023 | Singh V | Excluded | Evaluates patients who did not respond to terlipressin |
| 1. Role of terlipressin in patients with hepatorenal syndrome-acute kidney injury admitted to the ICU: a substudy of the CONFIRM trial | 2023 | Karvellas CJ | Excluded | Does not evaluate comparative efficacy of terlipressin and norepinephrine |
| 1. Role of terlipressin in cirrhotic patients with ascites and without hepatorenal syndrome: a systematic review of current evidence | 2020 | Bai Z | Excluded | Meta-analysis |
| 1. Noradrenaline or terlipressin for hepatorenal syndrome? | 2015 | Celis P | Excluded | Not an RCT |
| 1. Vasoconstrictors in hepatorenal syndrome – a critical review | 2019 | De Mattos AZ | Excluded | Not an RCT |
| 1. Comparison of terlipressin plus human albumin with norepinephrine plus human albumin in hepatorenal syndrome | 2022 | Ullah N | Excluded | Does not include HRS reversal as an endpoint |
| 1. Terlipressin in combination with albumin as a therapy for hepatorenal syndrome in patients with aged 65 years or older | 2023 | Mujtaba MA | Excluded | Does not evaluate comparative efficacy of terlipressin and norepinephrine |
| 1. Terlipressin versus placebo or no intervention for people with cirrhosis and hepatorenal syndrome | 2017 | Allegretti AS | Excluded | Does not evaluate comparative efficacy of terlipressin and norepinephrine |
| 1. The effect of terlipressin on renal replacement therapy in patients with hepatorenal syndrome | 2023 | Velez JCQ | Excluded | Does not evaluate comparative efficacy of terlipressin and norepinephrine |
| 1. Terlipressin versus noradrenaline for hepatorenal syndrome. Economic evaluation under the perspective of the Brazilian public health system | 2016 | De Mattos AZ | Excluded | Does not evaluate HRS reversal as an endpoint |
| 1. Treatment for hepatorenal syndrome in people with decompensated liver cirrhosis: a network meta-analysis | 2019 | Best LMJ | Excluded | Meta-analysis |
| 1. The current management of hepatorenal syndrome‐acute kidney injury in the United States and the potential of terlipressin | 2021 | Flamm SL | Excluded | Not an RCT |
| 1. Terlipressin reduces mortality in hepatorenal syndrome | 2016 | Maiwall R | Excluded | Not an RCT |
| 1. Safety and efficacy of terlipressin in acute-on-chronic liver failure with hepatorenal syndrome-acute kidney injury (HRS-AKI): a prospective cohort study | 2022 | Kulkarni A | Excluded | Does not evaluate comparative efficacy of terlipressin and norepinephrine |
| 1. Role of terlipressin and albumin for hepatorenal syndrome in liver transplantation | 2020 | Sharma P | Excluded | Does not evaluate comparative efficacy of terlipressin and norepinephrine |
| 1. Results of pretransplant treatment of hepatorenal syndrome with terlipressin | 2013 | Sola E | Excluded | Focuses on pretransplant treatment; does not evaluate comparative efficacy of terlipressin and norepinephrine |
| 1. Prognosis of acute kidney injury and hepatorenal syndrome in patients with cirrhosis: a prospective cohort study | 2015 | Allegretti AS | Excluded | Does not evaluate comparative efficacy of terlipressin and norepinephrine |
| 1. Terlipressin in hepatorenal syndrome | 2011 | Mazur JE | Excluded | Meta-analysis |
| 1. Hepatorenal syndrome: current concepts related to diagnosis and management | 2016 | De Mattos AZ | Excluded | Not an RCT |
| 1. Systematic review with meta-analysis: vasoactive drugs for the treatment of hepatorenal syndrome type 1 | 2016 | Gifford FJ | Excluded | Meta-analysis |
| 1. Hepatorenal syndrome: the clinical impact of vasoactive therapy | 2017 | Colle I | Excluded | Not an RCT |
| 1. The use of terlipressin in hepatorenal syndrome | 2010 | Rozov-Ung I | Excluded | Does not evaluate comparative efficacy of terlipressin and norepinephrine |
| 1. Terlipressin versus noradrenaline in the treatment of hepatorenal syndrome: systematic review with meta-analysis and full economic evaluation | 2015 | De Mattos AZ | Excluded | Meta-analysis |
| 1. Comparative efficacy of pharmacological strategies for management of type 1 hepatorenal syndrome: a systematic review and network meta-analysis | 2016 | Facciorusso A | Excluded | Meta-analysis |
| 1. Terlipressin for hepatorenal syndrome-acute kidney injury in acute-on-chronic liver failure: a single-center retrospective cohort study | 2021 | Liu S | Excluded | Retrospective study |
| 1. Efficacy of terlipressin and albumin in the treatment of hepatorenal syndrome | 2011 | Devrajani BR | Excluded | Does not evaluate comparative efficacy of terlipressin and norepinephrine |
| 1. Terlipressin is superior to noradrenaline in the management of acute kidney injury in acute-on-chronic liver failure | 2018 | Arora V | Included | Compares efficacy of terlipressin and norepinephrine amongst patients with HRS-1 with HRS reversal as a primary endpoint |
| 1. Hepatorenal syndrome: examine the treatment effectiveness of terlipressin and albumin | 2022 | Khan ZA | Excluded | Does not evaluate comparative efficacy of terlipressin and norepinephrine |
| 1. Noradrenaline vs terlipressin in the treatment of hepatorenal syndrome: a randomized study | 2012 | Singh V | Included | Compares efficacy of terlipressin and norepinephrine amongst patients with HRS-1 with HRS reversal as a primary endpoint |
| 1. Secondary scrotal necrosis to terlipressin treatment in a patient with hepatorenal syndrome | 2019 | Karakus V | Excluded | Does not evaluate comparative efficacy of terlipressin and norepinephrine |
| 1. Severe reversible penile ischaemia after terlipressin treatment of hepatorenal syndrome | 2018 | Lange LB | Excluded | Does not evaluate comparative efficacy of terlipressin and norepinephrine |
| 1. Hepatorenal syndrome: pathophysiology, diagnosis, and management | 2020 | Gines P | Excluded | Not an RCT |
| 1. Study of the effect of terlipressin with albumin vs only albumin in patients diagnosed with hepatorenal syndrome (HRS) | 2022 | Yeleshwaram VR | Excluded | Does not evaluate comparative efficacy of terlipressin and norepinephrine |
| 1. Early treatment with terlipressin in patients with hepatorenal syndrome yields improved clinical outcomes in North American studies | 2023 | Curry M | Excluded | Does not evaluate comparative efficacy of terlipressin and norepinephrine |
| 1. Hepatorenal syndrome | 2018 | Shredi A | Excluded | Not an RCT |
| 1. Hepatorenal syndrome | 2023 | Belcher JM | Excluded | Not an RCT |
| 1. A prospective study to compare the efficacy of noradrenaline verses terlipressin in hepatorenal syndrome in patients with advanced cirrhosis | 2021 | Nayyar S | Excluded | Compares efficacy of terlipressin and norepinephrine amongst patients with HRS-1 but there was no measurable effect |
| 1. Respiratory events with terlipressin and albumin in hepatorenal syndrome: a review and clinical guidance | 2022 | Allegretti AS | Excluded | Does not evaluate comparative efficacy of terlipressin and norepinephrine |
| 1. IDDF2021-ABS-0034 midodrine as a secondary prophylaxis for hepatorenal syndrome | 2021 | Parikh P | Excluded | Does not evaluate comparative efficacy of terlipressin and norepinephrine |
| 1. Noradrenaline versus terlipressin in the management of type 1 hepatorenal syndrome: a randomized controlled study | 2018 | Saif RU | Included | Compares efficacy of terlipressin and norepinephrine amongst patients with HRS-1 with HRS reversal as a primary endpoint |
| 1. Noradrenaline is as effective as terlipressin in hepatorenal syndrome type 1: a prospective, randomized trial | 2016 | Goyal O | Included | Compares efficacy of terlipressin and norepinephrine amongst patients with HRS-1 with HRS reversal as a primary endpoint |
| 1. Low doses of terlipressin and albumin in type I hepatorenal syndrome | 2008 | Pulvirenti D | Excluded | Does not evaluate comparative efficacy of terlipressin and norepinephrine |
| 1. Hepatorenal syndrome in patients with acute alcoholic hepatitis | 2012 | Jarcuska P | Excluded | Does not evaluate comparative efficacy of terlipressin and norepinephrine |
| 1. Terlipressin versus norepinephrine in the treatment of hepatorenal syndrome: a systematic review and meta-analysis | 2014 | Nassar AP Jr | Excluded | Meta-analysis |
| 1. Hepatorenal syndrome | 2022 | Cardenas A | Excluded | Not an RCT |
| 1. Terlipressin in the treatment of hepatorenal syndrome | 2014 | Zorniak M | Excluded | Not an RCT |
| 1. Hepatorenal syndrome | 2014 | Kiser TH | Excluded | Not an RCT |
| 1. Terlipressin and the treatment of hepatorenal syndrome: how the CONFIRM trial moves the story forward | 2021 | Belcher JM | Excluded | Not an RCT |
| 1. Vasoconstrictor therapy for hepatorenal syndrome | 2011 | Yeo CM | Excluded | Not an RCT |
| 1. Terlipressin and albumin for  type 1 hepatorenal syndrome associated with sepsis | 2014 | Rodriguez E | Excluded | Dual-diagnosis of HRS and sepsis; does not compare terlipressin with norepinephrine |
| 1. Pharmacokinetic and pharmacodynamic analyses of terlipressin in patients with hepatorenal syndrome | 2022 | Wang X | Excluded | Does not evaluate comparative efficacy of terlipressin with norepinephrine |
| 1. Hepatorenal syndrome | 2012 | Zeyneloglu P | Excluded | Not an RCT |
| 1. Terlipressin and albumin in patients with cirrhosis and type I hepatorenal syndrome | 2008 | Neri S | Excluded | Does not evaluate comparative efficacy of terlipressin with norepinephrine |
| 1. A study on clinical outcomes of combination of terlipressin and albumin in hepatorenal syndrome | 2020 | Krishna R | Excluded | Does not evaluate comparative efficacy of terlipressin with norepinephrine |
| 1. New developments in hepatorenal syndrome | 2017 | Mindikoglu AL | Excluded | Not an RCT |
| 1. Hepatorenal syndrome | 2022 | Gupta MM | Excluded | Not an RCT |
| 1. Albumin treatment regimen for type 1 hepatorenal syndrome: a dose-response meta-analysis | 2015 | Salerno F | Excluded | Meta-analysis |
| 1. Response to terlipressin and albumin is associated with improved liver transplant outcomes in patients with hepatorenal syndrome | 2020 | Piano S | Excluded | Does not evaluate comparative efficacy of terlipressin with norepinephrine |
| 1. Acute kidney injury and hepatorenal syndrome in cirrhosis | 2021 | Gupta K | Excluded | Not an RCT |
| 1. Terlipressin and albumin vs albumin in patients with cirrhosis and hepatorenal syndrome: a randomized study | 2008 | Martin-Llahi M | Excluded | Does not evaluate comparative efficacy of terlipressin with norepinephrine |
| 1. Terlipressin in hepatorenal syndrome: a systematic review and meta-analysis | 2010 | Dobre M | Excluded | Meta-analysis |
| 1. Prevalence and short-term outcome of hepatorenal syndrome: a 9-year experience in a high-complexity hospital in Colombia | 2020 | Rey M | Excluded | Retrospective study; not an RCT |
| 1. Hepatorenal syndrome: diagnostic and therapeutic management | 2016 | Bakulin IG | Excluded | Not an RCT |
| 1. Reduction in acute kidney injury stage predicts survival in patients with type 1 hepatorenal syndrome | 2020 | Wong F | Excluded | Does not evaluate comparative efficacy of terlipressin with norepinephrine |
| 1. Recent advances in the understanding and management of hepatorenal syndrome | 2021 | Simbrunner B | Excluded | Not an RCT |
| 1. Hepatorenal syndrome in metastatic cancers | 2022 | Rashidi A | Excluded | Does not include patients with HRS |
| 1. Terlipressin for hepatorenal syndrome: a meta-analysis of randomized trials | 2009 | Fabrizi F | Excluded | Meta-analysis |
| 1. Midodrine in the prevention of hepatorenal syndrome type 2 recurrence: a case-control study | 2009 | Alessandria C | Excluded | Does not include patients with HRS |
| 1. Clinical practice of hepatorenal syndrome: a brief review on diagnosis and management | 2021 | Rendy | Excluded | Not an RCT |
| 1. Terlipressin-induced skin necrosis while managing hepatorenal syndrome: a rare case report from North India | 2023 | Sudan S | Excluded | Not an RCT |
| 1. Hepatorenal syndrome with acute renal failure in patients with chronic liver disease: modern aspects of clinical presentation and intensive care | 2018 | Nagimullin RR | Excluded | Not an RCT |
| 1. Renal failure in patients with cirrhosis: hepatorenal syndrome and renal support strategies | 2010 | Meltzer J | Excluded | Not an RCT |
| 1. Hepatorenal syndrome | 2021 | Gill A | Excluded | Not an RCT |
| 1. Frequency of hepatorenal syndrome among patients with cirrhosis and outcome after treatment | 2020 | Fida S | Excluded | Does not evaluate comparative efficacy of terlipressin with norepinephrine |
