# Supplementary material for: Comparative efficacy of terlipressin and norepinephrine for treatment of hepatorenal syndrome-acute kidney injury: A systematic review and meta-analysis
Source: PLoS One. 2024 Jan 29;19(1):e0296690. doi: 10.1371/journal.pone.0296690 (PMC10824429; doi:10.1371/journal.pone.0296690)
Supplement: S2 Table — (DOCX) [file pone.0296690.s004.docx]

**Supplementary Table S2.** **Review authors' judgements about each risk of bias item using Cochrane risk of bias assessment**

Domain 1: Risk of bias arising from the randomization process; domain 2: Risk of bias due to deviations from the intended interventions (effect of assignment to intervention); domain 3: Missing outcome data; domain 4: Risk of bias in measurement of the outcome; domain 5: Risk of bias in selection of the reported result.

|  | Reviewer 1 | Reviewer 2 |
| --- | --- | --- |
| Alessandria et al [15] |  |  |
| Domain 1 | Some concerns | Some concerns |
| Domain 2 | High | Some concerns |
| Domain 3 | Low | Low |
| Domain 4 | Low | Low |
| Domain 5 | Low | Low |
| Overall risk of bias assessment | Some concerns | Some concerns |
| Sharma et al [17] |  |  |
| Domain 1 | Some concerns | Some concerns |
| Domain 2 | High | Some concerns |
| Domain 3 | Low | Low |
| Domain 4 | Low | Low |
| Domain 5 | Low | Low |
| Overall risk of bias assessment | Low | Low |
| Singh et al [18] |  |  |
| Domain 1 | High | Some concerns |
| Domain 2 | High | Some concerns |
| Domain 3 | Low | Low |
| Domain 4 | Low | Low |
| Domain 5 | Low | Low |
| Overall risk of bias assessment | Some concerns | Some concerns |
| Indrabi et al [19] |  |  |
| Domain 1 | Some concerns | Some concerns |
| Domain 2 | High | High |
| Domain 3 | High | High |
| Domain 4 | High | Low |
| Domain 5 | Some concerns | Some concerns |
| Overall risk of bias assessment | High | High |
| Goyal et al [16] |  |  |
| Domain 1 | Some concerns | Some concerns |
| Domain 2 | High | Some concerns |
| Domain 3 | Low | Low |
| Domain 4 | Low | Low |
| Domain 5 | Some concerns | Low |
| Overall risk of bias assessment | Some concerns | Some concerns |
| Saif et al [14] |  |  |
| Domain 1 | Some concerns | Some concerns |
| Domain 2 | High | Some concerns |
| Domain 3 | Low | Low |
| Domain 4 | Low | Low |
| Domain 5 | Low | Low |
| Overall risk of bias assessment | Some concerns | Low |
| Arora et al [12] |  |  |
| Domain 1 | Low | Low |
| Domain 2 | Low | Low |
| Domain 3 | Low | Low |
| Domain 4 | Low | Low |
| Domain 5 | Low | Low |
| Overall risk of bias assessment | Low | Low |
